# The E3 ligase subunit FBXO45 binds the interferon-λ receptor and promotes its degradation during influenza virus infection

**DOI:** 10.1016/j.jbc.2022.102698

**Published:** 2022-11-13

**Authors:** MuChun Tsai, Wissam Osman, Jessica Adair, Rabab ElMergawy, Lexie Chafin, Finny Johns, Daniela Farkas, Ajit Elhance, James Londino, Rama K. Mallampalli

**Affiliations:** Pulmonary, Critical Care and Sleep Medicine Division, The Ohio State University Wexner Medical Center, Columbus, Ohio, USA

**Keywords:** E3 ubiquitin ligase, influenza, interferon, IFNLR1, ubiquitination, F-box protein, BafA1, Bafilomycin A1, CHX, cycloheximide, FBS, fetal bovine serum, HIS, histine, IFN, interferon, IFNLR1, interferon lambda receptor 1, IL, interleukin, ISG, interferon stimulated genes, JAK, Janus kinase, NEM, N-ethylmaleimide, SCF, Skp-Cullin1-F- box, UPS, ubiquitin-proteasome system

## Abstract

Influenza remains a major public health challenge, as the viral infection activates multiple biological networks linked to altered host innate immunity. Following infection, IFN-λ, a ligand crucial for the resolution of viral infections, is known to bind to its cognate receptor, IFNLR1, in lung epithelia. However, little is known regarding the molecular expression and regulation of IFNLR1. Here, we show that IFNLR1 is a labile protein in human airway epithelia that is rapidly degraded after influenza infection. Using an unbiased proximal ligation biotin screen, we first identified that the Skp-Cullin-F box E3 ligase subunit, FBXO45, binds to IFNLR1. We demonstrate that FBXO45, induced in response to influenza infection, mediates IFNLR1 protein polyubiquitination and degradation through the ubiquitin-proteasome system by docking with its intracellular receptor domain. Furthermore, we found ectopically expressed FBXO45 and its silencing in cells differentially regulated both IFNLR1 protein stability and interferon-stimulated gene expression. Mutagenesis studies also indicated that expression of a K319R/K320R IFNLR1 variant in cells exhibited reduced polyubiquitination, yet greater stability and proteolytic resistance to FBXO45 and influenza-mediated receptor degradation. These results indicate that the IFN-λ–IFNLR1 receptor axis is tightly regulated by the Skp-Cullin-F box ubiquitin machinery, a pathway that may be exploited by influenza infection as a means to limit antiviral responses.

The RNA virus influenza robustly activates the immune system through molecular sensing of the viral RNA by host cells *via* pattern recognition receptors (Toll-like receptor, retinoic acid-inducible gene (RIG-I)), sequential activation of the mitochondrial antiviral-signaling protein signaling cascade, transcriptional activation of the DNA-binding component interferon (IFN) regulatory factors and NF-κB, and then type I (*e.g.*, IFN-α) and type III interferon (IFN-λ) production (1). IFN-λ is critical in the resolution of viral, bacterial, and fungal infections and acts through engagement of its cognate receptor complex, comprised of interferon lambda receptor 1 (IFNLR1) and interleukin (IL)-10R2 (2, 3). Four IFN-λ ligands (type III IFN) have been described with IFNλ1–3 sharing high amino acid sequence homologies, whereas IFNλ4 is more divergent with only 40.8% amino acid similarity to IFNλ3 (4, 5). Only IFNλ2 and 3 are conserved in mice. IFNLR1 (also known as IL-28R) is a single pass 520 AA type I membrane receptor. Upon ligand activation, STAT1 and STAT2 are recruited by IFNLR1 and IL-10rb, respectively, which are then phosphorylated and transported to the nucleus to mediate transcriptional activation of interferon-stimulated genes (ISGs) (4). IFNLR1 expression is largely restricted to mucosal surfaces such as the lung and gut epithelial layer in mice with a notable exception being high IFNLR1 expression in neutrophils (4). Several studies have shown that mice treated with IFN-λ after influenza infection exhibited significantly lower mortality, decreased viral burden, with reduced inflammatory cytokines compared to control underscoring the indispensability of the IFN-λ–IFNLR1 axis (6, 7). Further, the requirement of the IFNλ-IFNLR1 pathway for antiviral protection is underscored given that targeted disruption of IFNLR1 results in widespread viral dissemination and lethality (7).

In human tissues, IFNLR1 has a more diverse expression profile, but notably, the expression, signaling, and molecular regulation of IFNLR1 in the human lung has not been fully elucidated. In humans, IFN-λ is the earliest and most profuse interferon produced during influenza infection, triggering its engagement with IFNLR1 in providing host innate immunity (6, 8). Like rodents, IFN-λ may partake in antiviral defense at barrier surfaces, such as within the human lung and gastrointestinal epithelial lining. Remarkably, although both IFN-λ and IFN-α confer antiviral protection, IFN-α leads to the upregulation of proinflammatory genes (6). Multiple immune cells, including bone marrow–derived macrophages, dendritic cells, and neutrophils, stimulated with IFN-α, but not IFN-λ, secrete proinflammatory cytokines (6, 9). IFN-λ interestingly has been observed to inhibit several inflammatory mechanisms including ROS production, granule mobilization, and the release of neutrophil extracellular traps (10, 11). The IFNLR1 gene is robustly induced after influenza infection in humans, predicting interferon signaling (12).

Viral pathogenesis has been shown to exploit host cellular pathways for numerous steps of the infection cycle including molecular hijacking of the host protein degradation apparatus (13). The ubiquitin-proteasome system (UPS), executed *via* actions of E1-activating, E2-conjugating enzymes, and E3-ubiquitin ligases, are central to intracellular protein degradation. The UPS is fundamental to control of numerous cell functions involving protein turnover, cellular sorting and cell cycle progression, stress responses, transcriptional control, and surface receptor turnover (14, 15). Of the many E3 ligases, the understanding of Skp-Cullin1-F- box (SCF) superfamily is growing, comprised of a catalytic core consisting of Skp1, Cullin1, and Rbx1 (16, 17). The SCF complex also contains an adaptor receptor subunit, termed F-box protein, that engages numerous substrates to the E3 catalytic core (18, 19). F-box proteins are categorized within three families (FBXW, FBXO, FBXL) according to their substrate-binding motifs with the FBXO family containing a variety of yet unknown substrate-binding motifs (20). In particular, F-box proteins have been shown to partake in host defense in the pathogenesis of influenza infection, as FBXW7 antagonizes viral replication perhaps *via* RIG-I stabilization (21, 22). The FBXO family F-box protein, FBXO45, has been previously recognized to play a role in neoplasia, nervous system and psychiatric disorders, and inflammatory disorders (23). However, a biological role of this F-box protein remains largely unknown especially with respect to viral pathogenesis. In this study, we have observed that influenza infection triggers IFNLR1 degradation mediated by FBXO45 that targets IFNLR1 for ubiquitination and degradation in epithelia to impair IFN-λ signaling. The results suggest a potential mechanism whereby the virus subverts host immune responses essential for tissue repair.

## Results

### Influenza infection triggers IFNLR1 degradation *via* the ubiquitin-proteasome

**Figure 1:**
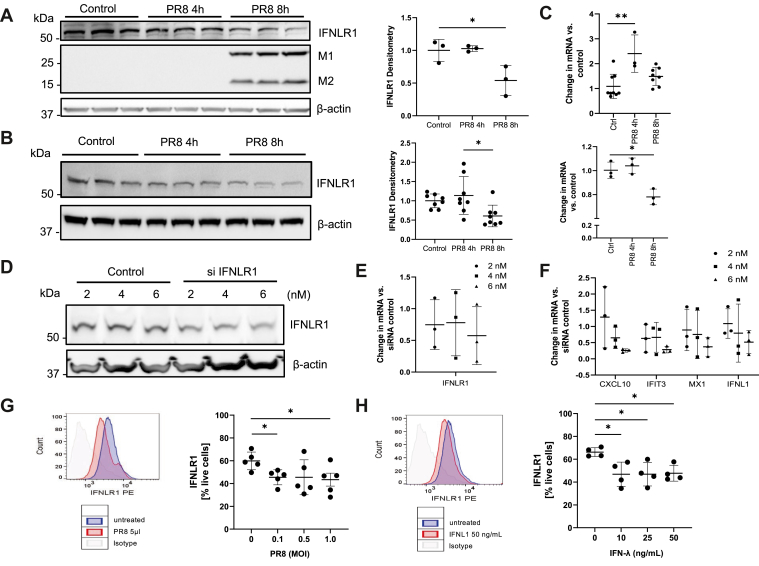
**Influenza rapidly degrades IFNLR1.***A* and *B*, THP-1 (*A*) differentiated for 7days in PMA (20 ng/ml) and BEAS-2B cells (*B*) were infected with influenza infection (PR8, MOI = 0.01) for 4 or 8 h before collection. IFNLR1, matrix 1 (M1), and Matrix 2 (M2) protein levels were detected by immunoblotting. The graphs on the right show IFNLR1 protein levels normalized to β-actin as quantitated using ImageJ software by densitometry. ∗*p* < 0.05 by one-way ANOVA. *C*, IFNLR1 mRNA levels were analyzed from (*A*). ∗*p* < 0.05, ∗∗ *p* < 0.01 by one-way ANOVA. *D*–*F*, BEAS-2B were transfected with either control or siRNA against IFNLR1 at various concentrations. Forty eight hours post transfection, cells were infected with PR8 (MOI = 0.05) and 24 h post infection, cells were processed for IFNLR1 immunoblotting (*D*) and mRNA (*E*) assay of interferon-stimulated genes (ISGs) by qPCR (*F*). *G*, IFNLR1 surface signals by flow cytometry significantly decreased after 6 h PR8 infection at different MOI’s in HBEC3KT cells. (*n* = 2) ∗*p* < 0.05 by unpaired, student’s *t* test. *H*, IFNLR1 decreased with treatment of recombinant IFN-λ to HBEC3KT cells at different concentrations for 6 h. (*n* = 2) ∗*p* < 0.05 by one-way ANOVA. Flow cytometry histograms on the *left* of (*G* and *H*) are representative from the HBEC3KT cells with indicated MOI of PR8 or concentrations of recombinant IFN-λ, respectively. All results were shown as mean ± SD. The data represent *n* = 3 separate experiments unless specified otherwise. IFNLR1, interferon lambda receptor 1.

We examined the short-term (<12 h) effect of influenza infection on IFNLR1 protein levels in THP-1 macrophages and BEAS-2B lung epithelial cells. Influenza infection significantly decreased IFNLR1 protein at 8 h post infection in both cell types (Fig. 1, *A* and *B*) without decreased receptor mRNA (Fig. 1*C*). We next examined the role of IFNLR1 signaling in BEAS-2B cells in transient knockdown studies using various amounts of siRNA to induce a modest reduction in IFNLR1 mRNA and protein. Here, despite modest knockdown of IFNLR1, we observed that levels of several antiviral genes were reduced in a trend toward a dose-dependent manner after PR8 infection (Fig. 1, *D–F*). Thus, the results demonstrate that epithelial cells are sensitive to availability of the IFNLR1 receptor that impact vital antiviral responses. To understand the upstream effect of influenza infection on IFNLR1 levels, we infected HBEC3KT cells, which are human telomerase reverse transcriptase-immortalized human bronchial lung epithelial cells. With increasing MOI of PR8 influenza infection of these cells over 6 h, we observed a significant decrease in IFNLR1 with PR8 infection *via* flow cytometry. (Fig. 1*G*). Next, we applied recombinant IFN-λ (IL-29) to HBEC3KT cells for 6 h at different concentrations. IFNLR1 signals *via* flow cytometry decreased with inclusion of IFN-λ (Fig. 1*H*) in the culture medium, suggesting that downregulation of IFNLR1 occurs due to autocrine signaling.

**Figure 2:**
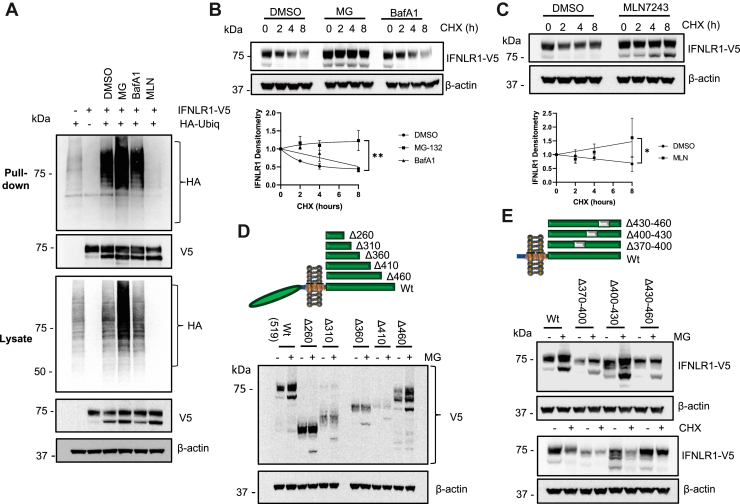
**IFNLR1 is polyubiquitinated and proteasomally degraded.***A*, HEK-293T cells were transfected with plasmids encoding V5- and HIS-tagged IFNLR1 and/or HA-tagged ubiquitin. Twenty four hours later, cells were treated with DMSO, MG-132 (20 μM), bafilomycin A1 (BafA1, 100 nM), or MLN (5 μM) for 4 h. Cell lysates were harvested, and the HIS-tagged IFNLR1 receptor was pulled down using magnetic cobalt beads and eluted with imidazole. Pull-down (above) and whole cell lysates (below) were immunoblotted to determine polyubiquitinated IFNLR1 (*n* = 2). *B* and *C*, HEK-293T cells were transfected with V5-tagged IFNLR1 plasmid for 24 h followed by pretreatment with DMSO, MG-132 (MG, 20 μM), or bafilomycin A1 (BafA1, 100 nM) or DMSO (*B*) or the E1 inhibitor MLN7243 (MLN, 5 μM) with CHX (50 μg/ml) at indicated time points. Shown graphically below are densitometric analysis of immunoblots. ∗*p* < 0.05 and ∗∗*p* < 0.01 by one-way ANOVA (*C*). IFNLR1 protein stability was analyzed by V5 immunoblotting. *D* and *E*, HEK-293T cells were transfected with V5-tagged IFNLR1 or V5-tagged IFNLR1 receptor plasmids with truncated C-terminal domains (*top schematic*) (*D*) or with V5-tagged IFNLR1 receptor plasmids with a series of 30 AA deletions (*E*). Twenty four hours later, cells were treated with DMSO or MG-132 (20 μM), and cell lysates were harvested for V5 immunoblotting. The data represent *n* = 3 separate experiments unless specified otherwise. CHX, cycloheximide; IFNLR1, interferon lambda receptor 1.

To understand the degradation process further, we next ectopically expressed V5- and histine (HIS)-tagged IFNLR1 and HA-tagged–ubiquitin in HEK-293T cells. This was followed by treatment with the proteasomal inhibitor MG-132 or autophagosome/lysosomal inhibitor Bafilomycin A1 (BafA1) to preserve the ubiquitinated proteins. MLN7243, an inhibitor of ubiquitin activating enzyme, was added as a control to confirm that the detected bands were due to ubiquitination. Cell lysates were harvested, and samples were processed for HIS-tagged pull-down assays to compare the differences in polyubiquitination. BafA1 did not significantly increase ubiquitination above basal levels, whereas MG-132 led to a robust increase in IFNLR1 polyubiquitination (Fig. 2*A*). Next, in cells treated with the protein synthesis inhibitor, cycloheximide (CHX), IFNLR1 protein was largely degraded by 8 h, an effect significantly blocked by inclusion of MG-132 and MLN7243 in the culture medium but not BafA1 (Fig. 2, *B* and *C*). To map the region of IFNLR1 protein necessary for degradation, we constructed plasmids expressing IFNLR1 with truncated C-terminal domains and after transfection into BEAS-2B cells, we measured stabilization of each variant by MG-132 (Fig. 2*D*). When truncations expressed in cells do not accumulate in the presence of MG-132, this suggests the loss of regions necessary for degradation. Although these constructs expressed to variable degrees in cells, our data showed that both the WT full-length protein and a construct harboring the first 460 AA of the IFNLR1 protein showed accumulation after MG-132 unlike other truncations (Fig. 2*D*). These data suggest the presence of molecular signatures spanning residues AA 360-460 that may be required for receptor degradation. To examine this region more closely, we constructed a series of 30 AA deletions between the AA 370-460 domain. When AA 430-460 was excised and the plasmid expressed in cells, the resulting protein was both less responsive to MG-132 and more stable in the presence of CHX (Fig. 2*E*). Using protein motif prediction software (http://emboss.bioinformatics.nl/cgi-bin/emboss/epestfind), we identified that this region corresponds to a PEST domain (AA 432–486), a canonical destabilizing element (24). Together, the data suggest that IFNLR1 is (i) ubiquitinated and proteasomally degraded and (ii) that there might reside a specific destabilizing motif necessary for degradation within the cytoplasmic domain.

### FBXO45 binds IFNLR1 to modulate interferon signaling

**Figure 3:**
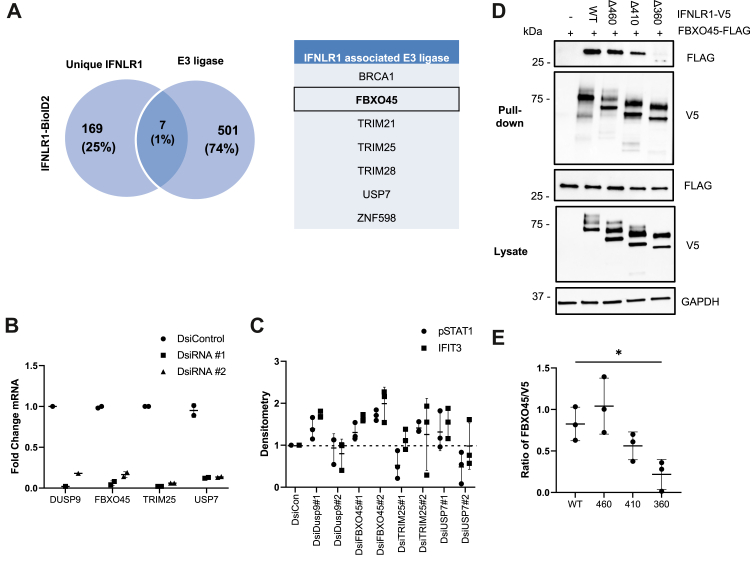
**FBXO45 targets IFNLR1 for degradation.***A*, BioID2 biotin ligase was conjugated separately to the C-terminus of IFNLR1. We overexpressed BioID2 alone, the interferon gamma receptor IFNGR1-BioID2, or the transmembrane receptor SIRPA-BioID2 as specificity controls. HEK-293T cells were transiently transfected with these constructs for 48 h followed by 1 h biotin treatment and isolation of total protein. We identified 169 unique IFNLR1 proteins, 7 of which were ubiquitin E3 ligases, including FBXO45. *B*, several IFNLR1-associated E3 ligase proteins, (FBXO45, TRIM25, and USP7), in addition to diphosphate DUSP9, were effectively knocked down in BEAS-2B cells with two different DsiRNA and levels of mRNAs encoding these proteins assayed by qPCR. *C*, BEAS-2B cells were transfected with indicated DsiRNA. Forty eight hours later, cells were treated with IFNλ-1 (20 ng/μl) for 30 min or 4 h to measure STAT1 phosphorylation or IFIT3 induction. The graph shows the fold change in protein levels after densitometric analysis of pSTAT1 and IFIT3 immunoblots. *D*, HEK-293T cells were cotransfected with FLAG-tagged FBXO45 and either V5-tagged IFNLR1-WT or five different truncated V5-IFNLR1 mutants. After 48 h, cells were treated with MG-132 (20 μM) and leupeptin (100 μM) for 2 h. Cell lysates were harvested and a co-IP V5 pull-down was performed. Pull-down (above) and whole cell lysate (below) were immunoblotted to determine binding affinity between FBXO45 and the IFNLR1 mutants (*n* = 2). *E*, the graph shows relative IFNLR1 protein level interaction with FBXO45 normalized to β-actin as quantitated using ImageJ software by densitometry. ∗*p* < 0.05 by one-way ANOVA. Results were shown as mean ± SD. Data represent *n* = 3 separate experiments. IFNLR1, interferon lambda receptor 1.

We used BioID2 and miniTurbo proximity ligation assays as unbiased screens to determine molecular binding partners that might mediate IFNLR1 degradation. The BioID2 biotin ligase was conjugated to the carboxyl-terminus of IFNLR1. As specificity controls, we overexpressed the IFN-γ receptor IFNGR1-BioID2 and the transmembrane receptor SIRPA-BioID2. HEK-293T cells were transiently transfected with these constructs for 48 h followed by overnight biotin treatment and isolation of biotinylated proteins. We identified 169 unique IFNLR1 proteins, 7 of which were E3 ligases, including FBXO45 (Fig. 3*A*). In separate experiments, we also conjugated the biotin ligase miniTurbo to the carboxyl-terminus of IFNLR1, transiently transfected into cells, and treated cells with biotin for 1 h prior to harvest. Compared to miniTurbo alone, we identified 100 unique interactions with the IFNLR1-miniTurbo biotin ligase. Six of these proteins were E3 ligases. FBXO45 was again identified as a unique IFNLR1-interacting partner (Fig. S1). Due to our identification of an IFNLR1–FBXO45 interaction in multiple experiments using different biotin ligase constructs, we further examined FBXO45-mediated changes in IFNLR1 signaling. We examined several ubiquitin-related proteins uniquely associated with IFNLR1, including FBXO45, TRIM25, and USP7, as well as the diphosphate DUSP9 as a potential regulator of IFNLR1 function. Cellular depletion of all four genes using two different DsiRNA was executed in BEAS-2B cells that were transfected with these corresponding DsiRNA (Fig. 3*B*) and treated with IFN-λ. Here, we observed an increase in IFNLR1-mediated STAT1 phosphorylation and downstream IFIT3 gene induction with both DsiRNA’s targeting FBXO45 (Fig. 3*C*). Thus, we focused on FBXO45. To also map the location of FBXO45 and IFNLR1 protein interaction, we utilized a series of truncated IFNLR1 C-terminal domain proteins and performed coimmunoprecipitation experiments using the truncation mutants of V5-tagged IFNLR1 and FLAG-tagged FBXO45 to locate the receptor region that binds FBXO45. We found that the IFNLR1 constructs lacking the region between AA360 and 410 exhibited substantially reduced ability to bind to FBXO45, suggesting that there might exist a motif in this region necessary for FBXO45 docking (Fig. 3, *D* and *E*).

**Figure 4:**
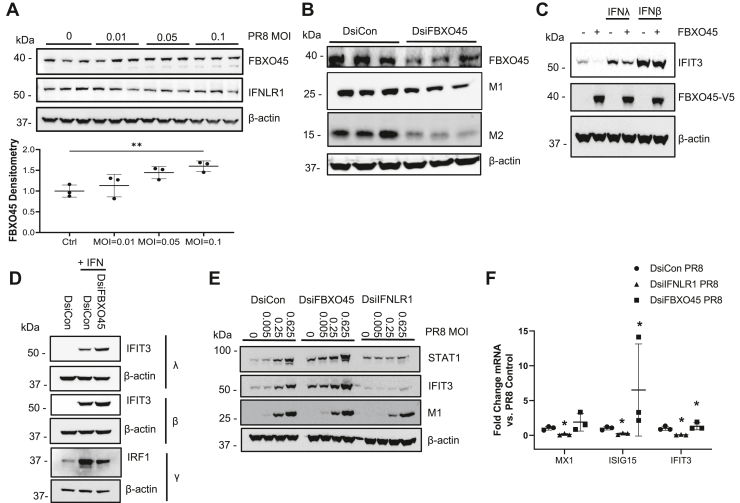
**FBXO45 is required and sufficient to modulate influenza activation of ISG.***A*, BEAS-2B cells were infected with PR8 at various MOI for 6 h and lysates were processed for endogenous FBXO45 and IFNLR1 levels by immunoblotting. The graph on the *bottom* shows FBXO45 protein levels normalized to β-actin as quantitated using ImageJ software by densitometry. ∗∗*p* < 0.01 by one-way ANOVA. *B*, THP-1 cells were differentiated for 24 h followed by transfection with 15 nM DsiCon or FBXO45 siRNA. At 48 h post siRNA transfection, cells were infected with PR8 (MOI = 0.1). Twenty four hours post infection, cell lysates were processed for FBXO45, matrix 1 (M1), and Matrix 2 (M2) immunoblotting. *C*, BEAS-2B cells were transfected with FBXO45 plasmid or an empty vector for 24 h followed by treatment with IFNλ-1 or IFN-β and IFIT3 immunoblotting. *D*, BEAS-2B cells were transfected with DsiRNA against V5-tagged FBXO45 for 48 h followed by IFN treatment and IFIT3/IRF1 immunoblotting. *E* and *F*, BEAS-2B cells were transfected with DsiRNA against FBXO45 or IFNLR1 and infected with PR8 for 8 h. After 48 h, protein (*E*) and RNA (*F*) was isolated and ISG induction was measured *via* immunoblotting and qRT-PCR, respectively. Results were shown as mean ± SD. ∗*p* < 0.05 *versus* control (DsiCon) by students *t* test. Results were shown as mean ± SD. Data represent *n* = 3 separate experiments. IRF, IFN regulatory factor; IFNLR1, interferon lambda receptor 1; ISG, interferon-stimulated gene.

Additional studies were conducted to assess viral responses of FBXO45 in cells and effects of the F-box protein on interferon signaling. In BEAS-2B cells, increasing MOI’s of PR8 influenza increased FBXO45 levels coupled with a reduction in levels of IFNLR1 (Fig. 4*A*). Interestingly, FBXO45 silencing and subsequent PR8 infection reduced matrix 1/matrix 2 (M1/M2) proteins (Fig. 4*B*). Further, FBXO45 overexpression in BEAS-2B cells selectively decreased IFN-λ signaling (Fig. 4*C*). When FBXO45 was depleted in cells and subsequently treated with IFN-λ, IFN-α, or IFN-γ, only IFN-λ induced IFIT3 after FBXO45 knockdown (Fig. 4*D*). Infection of BEAS-2B cells with various MOI of PR8 and FBXO45 silencing also led to a substantial increase in ISG expression, an effect not observed after IFNLR1 cellular depletion (Fig. 4*E*). Specifically, the proteins STAT1 and IFIT3 were notably increased in a dose-dependent manner compared to the control with FBXO45 silenced. Last, the antiviral genes ISG15 and IFIT3 mRNAs were also increased with knockdown of FBXO45 but reduced with IFNLR1 silencing. Together, these data suggest that FBXO45 plays a key role in attenuating antiviral host defense to facilitate influenza virulence.

### FBXO45 destabilizes IFNLR1 protein

**Figure 5:**
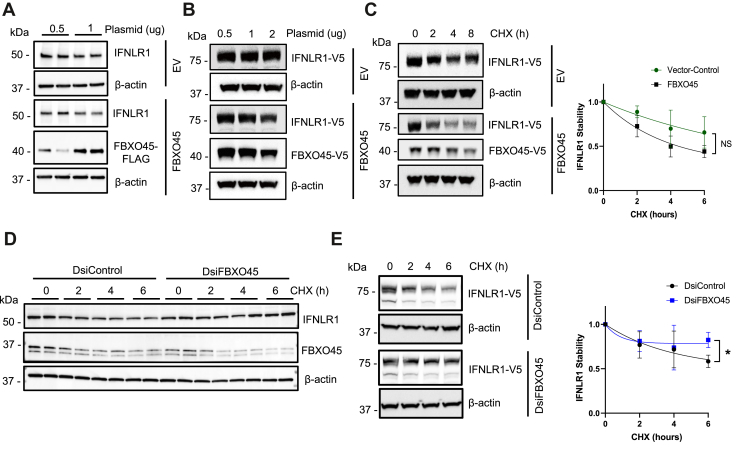
**FBXO45 decreases IFNLR1 stability.***A*, BEAS-2B cells were transfected with either empty vector (EV) or FBXO45-V5 plasmid at indicated concentrations for 48 h and cells processed for endogenous IFNLR1 and FBXO45-FLAG (*n* = 2) and shown are levels of endogenous IFNLR1, actin, and FBXO45. *B*, BEAS-2B cells were cotransfected with V5-tagged IFNLR1 plasmid and either FBXO45-V5 or empty vector (EV) at indicated concentrations for 48 h and cells processed for IFNLR1-V5 and FBXO45-V5 immunoblotting. *C*, BEAS-2B cells were cotransfected as in (*B*), followed by a CHX (50 μg/ml) chase at indicated time points and cells processed for IFNLR1-V5 and FBXO45-V5 immunoblotting. The graph on the *right shows* decay of the IFNLR1 protein over time after densitometric analysis of immunoblots. *D*, BEAS-2B were treated with control siRNA or FBXO45 siRNA for 48 h followed by a CHX (50 μg/ml) chase at indicated time points. Cells were processed for immunoblotting of endogenous IFNLR1 and FBXO45 levels (*n* = 2). *E*, BEAS-2B cells were transfected with V5-tagged IFNLR1 plasmid and either control siRNA or FBXO45 siRNA for 72 h followed by a CHX (50 μg/ml) chase at indicated time points. IFNLR1 protein stability was assayed as in (*C*) and results shown graphically on right graph. ∗*p* < 0.05 *versus* control (DsiControl) at the 6 h timepoint by students *t* test. Results were shown as mean ± SD. Data represent *n* = 3 separate experiments unless specified otherwise. CHX, cycloheximide; IFNLR1, interferon lambda receptor 1.

**Figure 6:**
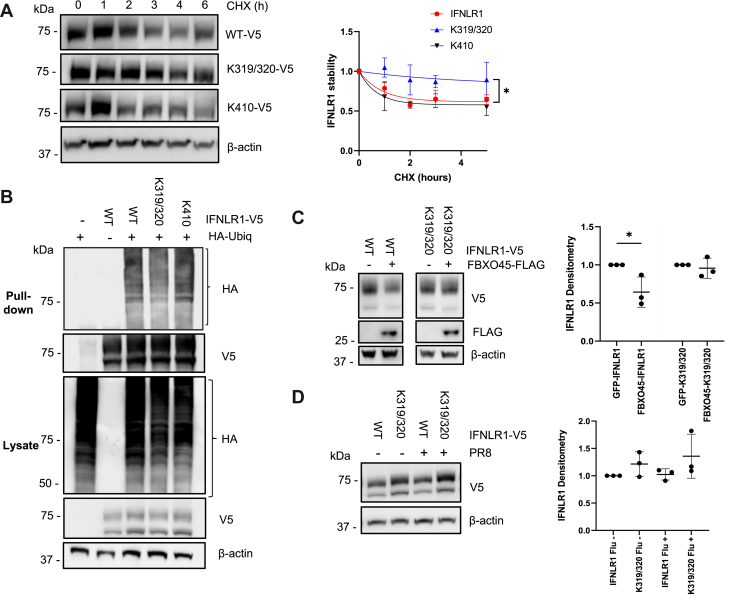
**Ectopically expressed IFNLR1 variant displays proteolytic resistance to influenza infection and FBXO45.***A*, HEK-293T cells transfected with V5-tagged IFNLR1 plasmid, IFNLR1-K319R/K320R, or IFNLR1-K410R for 48 h followed by a CHX (40 μg/ml) chase and cells processed for V5 immunoblotting. Shown on the right graph is decay of the V5-IFNLR1 protein over time after densitometric analysis of immunoblots. ∗*p* < 0.5 by one-way ANOVA. *B*, HEK-293T cells were transfected with V5-tagged IFNLR1, IFNLR1-K319R/K320R, or IFNLR1-K410R and HA-tagged ubiquitin. Forty eight hours later, cells were treated with MG-132 (20 μM) for 4 h. Cell lysates were harvested and a V5 pull-down was performed. Whole cell lysate (below) and pull-down (above) were immunoblotted to determine ubiquitinated products, (*n* = 2). *C*, HEK-293T cells were cotransfected with V5-tagged IFNLR1 or IFNLR1-K319R/K320R and FLAG-tagged FBXO45 *versus* GFP-tagged empty vector for 48 h and processed for V5 and FLAG immunoblotting. ∗*p* < 0.5 by students *t* test. *D*, HEK-293T cells were transfected with V5-tagged IFNLR1 or IFNLR1-K319R/K320R. After 48 h, the cells were infected with PR8 (MOI = 0.01) for 3 h and cells harvested for V5 immunoblotting. In (*C* and *D*), the graphs on the right show the relative levels of expressed proteins after densitometric quantitation of immunoblots. Results were shown as mean ± SD. Data represent *n* = 3 experiments unless specified otherwise. CHX, cycloheximide; IFNLR1, interferon lambda receptor 1.

Given the molecular interaction of FBXO45 with IFNLR1 and its ability to antagonize interferon signaling, we next assessed the F-box protein on its ability to modulate receptor protein degradation. We transfected BEAS-2B cells with FLAG-tagged FBXO45 plasmid or empty vector at 0.5 or 1.0 μg for 48 h and observed a reduction of endogenous IFNLR1 by ∼35% *versus* empty vector (Fig. 5*A*). We also transfected BEAS-2B cells with V5-tagged IFNLR1 plasmid and either V5-tagged FBXO45 or an empty vector at 0.5, 1, or 2 μg for 24 h and observed a dose-dependent reduction in steady-state IFNLR1 mass (Fig. 5*B*). When BEAS-2B cells were transfected as above with an empty vector or with a FBXO45-mutant plasmid (lacking E3 ligase catalytic activity) followed by a CHX chase, we found that WT FBXO45 overexpression accelerated IFNLR1 protein decay and tended to reduce its half-life (Fig. 5*C*), whereas there was no change in half-life with expression of a FBXO45-mutant *versus* an empty construct (Fig. S2). Conversely, when we silenced FBXO45 in BEAS-2B cells, we found increased endogenous IFNLR1 lifespan (Fig. 5*D*). Similar findings were observed when we transfected BEAS-2B cells with V5-tagged IFNLR1 and two separate DsiRNA’s targeting FBXO45 (Figs. 5*E* and S3). These results strongly implicate FBXO45 as an E3 ligase component that specifically regulates IFNLR1 protein turnover. To further assess the molecular signatures involved in IFNLR1 protein destabilization, we constructed IFNLR1 point mutants at candidate lysines within the cytoplasmic region: K319/320 (double site) and K410. After cellular expression, we analyzed IFNLR1-WT, IFNLR1-K319R/K320R, and IFNLR1-K410R t_1/2_ using CHX over 6 h (Fig. 6*A*). Notably, IFNLR1-K319R/K320R remained stable with a longer t_1/2_, whereas IFNLR1-WT and IFNLR1-K410R degraded rapidly over time. We then evaluated levels of polyubiquitination of these mutants by cotransfecting V5-tagged constructs and HA-tagged ubiquitin in HEK-293T cells followed by treatment with MG-132. Cell lysates were harvested with DUB inhibitors, 1,10-phenanthroline, PR-619, and N-ethylmaleimide (NEM). Polyubiquitination was evaluated by immunoprecipitation, which revealed greater signal intensity in IFNLR1-WT and IFNLR1-K410R in comparison to the IFNLR1-K319R/K320R variant (Fig. 6*B*). To inspect whether the IFNLR1 double mutant could resist degradation with the presence of FBXO45, HEK-293T cells were cotransfected with either V5-tagged IFNLR1-WT or IFNLR1-K319R/K320R and FLAG-tagged FBXO45 or GFP-tagged empty vector. Here, the IFNLR1-K319R/K320R was stable despite ectopic expression of FBXO45 (Fig. 6*C*). Finally, because influenza infection increased IFNLR1 degradation, we evaluated the stability of the IFNLR1-K319R/K320R variant after PR8 infection for 3 h. The data show that IFNLR1-K319R/K320R was more resistant to viral-induced degradation than IFNLR1-WT (Fig. 6*D*). Thus, the results suggest that degradation of IFNLR1 occurs *via* polyubiquitination *via* the SCF^FBXO45^ E3 ligase, possibly at the K319/K320 molecular site(s) during influenza infection.

## Discussion

In this study, we demonstrate for the first time that IFNLR1 is potentially a new substrate for the E3 ligase component FBXO45 that may modulate viral pathogenesis. IFN-λ, acting *via* IFNLR1, has been shown to be important in the resolution of viral, bacterial, and fungal infections (2, 3). IFNLR1 is predominately found on epithelial cells, which can provide localized antiviral protection along barrier surfaces. Studies have shown that early IFN-λ treatment in mice during influenza virus infection offers significant antiviral protection without the proinflammatory responses associated with Type 1 interferons (6, 25). Although we have previously demonstrated the presence of the IFN-λ–IFNLR1 signaling axis in human lung macrophages and its role in combating influenza infection, the expression, signaling, and molecular regulation of IFNLR1 in human bronchial epithelial cells has not been fully elucidated (26).

The new contributions in this study provide associations between influenza infection of epithelia, FBXO45, and its interaction with IFNLR1 triggering its degradation. The F-box protein is induced after viral infection and in an unbiased screen, FBXO45 binds IFNLR1 that appears to be localized to a region spanning 50 AA within the cytoplasmic domain. This region (AA 360–410) appears downstream of previously identified binding sites for human Janus kinase 1 (JAK1) that also binds the intracellular domain of IFNLR1 (27) and excludes IFN-λ1–induced STAT2 tyrosine phosphorylation sites within the receptor (28). The identification of phosphorylation sites within the cytoplasmic or soluble region of IFNLR1 may be important given that F-box proteins are recruited to substrates through phosphosite recognition. However, there may be other posttranslational modifications within this region such as glycosylation that have been described to enhance substrate–SCF complex engagement within the FBXO family (29, 30). Nevertheless, a precise mapping of binding motifs within the IFNLR1 cytoplasmic domain by FBXO45 will require additional analysis using recombinant interactors and perhaps structural studies.

We observed that IFNLR1 is relatively unstable in the native state and yet further depleted in lung epithelial cells during influenza infection through the UPS, specifically by the SCF^FBXO45^. The lifespan of the IFNLR1 receptor somewhat resembles that of the IFNγ receptor 1 that is also labile (t_½_ ∼ 2 h) (31). Interestingly, IL-22, similar to IFN-λ, is another member of the IL-10 cytokine family that functions at epithelial mucosal barriers in response to infection (32). Both IFN-λ and IL-22 signal through a group of heterodimeric receptors, class II cytokine receptors, their protein structures are highly related, and upon ligand engagement, they trigger canonical JAK/STAT activation to confer antiviral activities (33, 34). Of note, IL-22R, the cognate IL-22 receptor is targeted for ubiquitination by FBXW12 in human lung epithelia (32). In the present work, FBXO45 was sufficient and required to differentially affect IFNLR1 protein stability, the receptor was polyubiquitinated, and we uncovered at least two putative molecular acceptor sites within the cytoplasmic domain for ubiquitination using mutagenesis. We recognize that IFNLR1 polyubiquitination may also vary depending on the experimental context, and that other linkages (K63 *versus* K48) may occur exclusively or in combination within subpopulations of surface receptor molecules that govern receptor stability or its subcellular trafficking. Additionally, although only six cytosolic lysines are present within the receptor, there may be multiple acceptors or other E3 ligases that mediate IFNLR1 turnover. This appears to be the case with NLRP3, an intracellular pathogen sensor that is ubiquitinated by the E3 ligases gp78 and TRIM31 (35). Our observation that FBXO45 but other ubiquitin ligases were not captured in our unbiased screen coupled with loss-of-function and gain-of-function studies using FBXO45 provide preliminary evidence of its effector role in the IFN-λ–INFLR1 innate immunity complex.

The discovery of FBXO45 and its behavior on the IFN-λ–IFNLR1 axis may add a layer of complexity given the vast majority of other studies implicating the F-box protein as an oncogene (23). FBXO45 has been described as a tumor promoter or linked to the pathogenesis of pancreatic, esophageal, liver, gastric, lung, and breast carcinomas (23, 36, 37, 38, 39, 40, 41). Importantly, type III interferons such as IFN-λ have been shown to inhibit tumor cell proliferative activity and activate neoplastic cell apoptosis, perhaps *via* IFNLR1 ligation (42, 43, 44, 45, 46, 47). Hence, it is conceivable that within the tumor microenvironment, the concentrations of IFNLR1 are exquisitely controlled by FBXO45 such that the robustness of signaling events downstream of IFN-λ–receptor activation (JAK-STAT1) impacts tumorigenesis. On one hand, FBXO45 depletion of IFNLR1 and consequentially reduced JAK-STAT1 activation may result in reduced expression of antitumor genes. Alternatively, the observations that JAK-STAT inhibitors are currently in use in the clinical setting as antitumor therapeutics (48, 49) suggest that FBXO45 activation might interact with other yet unknown clients to induce cell proliferation linked to JAK-STAT1 activation that impact development of cancer. The findings of this study further align with the supposition that innate immunity and tumorigenesis are linked.

Previous studies have shown that IFN-λ promotes epithelial barrier integrity during viral infection models and not surprisingly IFN-λ has been considered as a therapeutic option for respiratory viral infections (50, 51). However, recent studies suggest that increased IFN-λ production during infection actually hinders lung epithelial repair in mice during influenza recovery (52). Other studies have demonstrated that exposure to IFN-λ impaired epithelial barrier function, increasing the susceptibility to bacterial infection (53, 54). While these studies regarding IFN-λ and its effect on the epithelial barrier are inconsistent, it may be due to differences in the infection model, timing of interferon application, and particular viruses studied. The identification of IFNLR1 as a substrate for FBXO45 as shown here might underlie the discrepancies in the bioactivity of IFN-λ; if indeed, concentrations of IFNLR1 are depleted by ubiquitin E3 ligases in one model system *versus* another. Our discovery of IFNLR1 degradation by FBXO45 may potentially provide some therapeutic applications as well as facilitate further research in the IFN-λ–IFNLR1 signaling pathway within lung epithelial cells.

## Experimental procedures

### Materials

#### Antibodies

Antibodies used in immunoblotting were as follows: anti-IFNLR1 (Sigma-Aldrich, HPA017319), anti-M1 (Abcam, ab22396), anti-M2 (Novus Biologicals, 14C2), anti-β-Actin (Sigma-Aldrich, A5441), anti-GAPDH (Cell Signaling Tech, 2118), anti-HA (Santa Cruz, sc-7392), anti-V5 antibody (CST, 13202S), anti-IFIT3 (Santa Cruz, sc-393512), anti-IRF (CST, 8478S), anti-pSTAT1 (CST, 7649), anti-FLAG antibody (Sigma-Aldrich, F3165), and anti-FBXO45 (Aviva Systems Biology, ARP 66799_P050). Interferons used are as follows: IFN-λ (R&D system, 1598-IL), IFN-β (Abcam, ab71475), and IFN-γ (BioLegend, 570206). Neutralizing antibody are as follows: anti-IL-29/IFN-λ1 (R&D systems, AF1598); normal goat IgG control (R&D systems, AB108C).

#### Cell cultures and reagents

Human bronchial epithelial BEAS-2B cells and human embryonic kidney 293 (HEK-293T) cells were purchased from the American Type Culture Collection. HEK-293T cells were cultured in Dulbecco’s modified Eagle’s medium (Gibco) supplemented with 10% fetal bovine serum (FBS) (GeminiBio). BEAS-2B cells were cultured in 10% HITES medium (Dulbecco’s modified Eagle’s medium/F12 (1:1, Gibco) supplemented with 0.5% ITS-G, (Gibco) 10 nM hydrocortisone, 10 nM β-estradiol, 0.1 mg/ml Transferrin, and 10% FBS (GeminiBio)).

#### Infection protocol

Influenza PR8 was propagated on MDCK cells (American Type Culture Collection, CCL-34) (55). Cells were infected at the indicated MOI for various times, and the media was replaced after 1-2 h.

#### Quantitative PCR

Total cellular RNA was collected from cells using the Qiagen RNeasy Miniprep plus Kit (Qiagen), following the manufacturer's protocol. The cellular RNA was then used to create complementary DNA (cDNA) using the High-Capacity cDNA Reverse Transcription Kit (Applied Biosystems) according to the manufacturer's protocol. Quantitative PCR was performed using SYBR Select Master Mix (Applied Biosystems) according to the manufacturer's protocol with 20 ng cDNA as a template and primer concentration of 200 nM. Each biological replicate was performed in at least technical duplicate; data was analyzed using the ΔΔCq method.

#### Immunoblotting

Procedures were performed as described previously (31). Briefly, cells were lysed in RIPA buffer (Sigma, R0278), sonicated, and clarified by centrifugation. Lysates were diluted in (Laemmli Sample Buffer, Bio-Rad) SDS protein sample buffer. Proteins were separated by electrophoresis on a 4 to 12% gel (Invitrogen) and transferred to a 0.22 um nitrocellulose membrane (Bio-Rad). Blots were blocked in 5% milk, followed by probing overnight with antibodies. Following addition of HRP-conjugated secondary antibodies (goat anti-mouse, Bio-Rad, 170–6516 and goat anti-rabbit, Bio-Rad, 170–6515; 1:2000 dilution), membranes were developed using a Western Bright Sirius immunoblotting detection kit (Advansta). Images were developed using a ChemiDoc Imaging System and ImageLab Touch software version 2.3 using a 15 s exposure of immunoblots. Single band intensity was quantified using ImageJ software.

#### Flow cytometry

HBEC3KT cells were removed from cell culture dishes using Accutase (ThermoFisher Scientific) and resuspended in 100μl 1×PBS. For viability, cells were incubated at room temperature for 10 min with 0.2 μl Zombie Aqua Fixable Viability stain (BioLegend) in 100 μl PBS. Control PE-mIgG2a, κ isotype (BioLegend, 400213), or PE-IL-28RA antibody (BioLegend, 337804) was added and incubated on ice for an additional 20 min. After incubation, the cells were washed with 1× PBS + 2% FBS and data were collected on a BD FACSymphony A1 Cell Analyzer (BD Biosciences) and analysis was performed using FlowJo software (FlowJo).

#### Protein half-life assay

Cells were evenly divided into 6- or 12-well plates and transfected with designated plasmids. Twenty four to forty eight hours after transfection, the cells were then treated with the protein synthesis inhibitor CHX (Cayman Chemical, 40–50 μg/ml) for the indicated times before collection. For the inhibitors, MG-132 (Fisher Scientific), bafilomycin (Sigma-Aldrich), and MLN7243 (Chemietek) were added at the same time as CHX.

#### Plasmids, cloning, and cell transfections

The pcDNA3.1/V5-His was purchased from Invitrogen and pRK5-HA-Ubiquitin-KO was purchased from Addgene. The V5- and HIS-tagged IFNLR1 construct utilized the pcDNA3.1 expression plasmid, under an Ef1α promoter. The mutants and target genes with different tags were cloned into the pcDNA3.1 vector using standard cloning procedures. Constructs were validated *via* sequencing. For overexpression, cells were transfected using XtremeGene transfection HP DNA reagents (Roche, 06366546) according to manufacturer’s protocol. DsiRNAs were all obtained from Integrated DNA Technologies. Cells were transfected with siRNA using GenMute siRNA Transfection reagent (SignaGen, SL100568) according to manufacturer’s protocol.

#### Proximity ligation biotin pull-down

The biotin ligase constructs, MCS-BioID2-HA (#74224) and V5-miniTurbo-NES_pCDNA3 (#107170), were obtained from Addgene and conjugated to the carboxyl-terminal of IFNLR1. HEK-293T were transfected with these constructs and then treated with 50 μM biotin (Fisher Scientific) for 1 h. Media was replaced with biotin-free media for the final 15 to 30 min. Cells were washed with cold 1× PBS on ice twice, and cells harvested with cold buffer (PBS with 1% Triton X-100, 0.2% SDS, and protease inhibitor (Thermo Scientific)). Samples were rotated at 4 °C for 20 min and then sonicated for 30 s at 25 °C three times. Streptavidin magnetic beads (Pierce) were magnetically sorted and rinsed with wash buffer 1 (PBS with 1% Triton X-100, 0.2% SDS) twice. Beads were then added to samples and rotated at 4 °C for 2 h. Beads were rinsed with wash buffer 1 twice, then rinsed with wash buffer 2 (8M urea in 50 mM Tris made in 1xPBS) twice more. Beads were rinsed again with wash buffer 1 then 1× PBS. Finally, 4× SDS + biotin mix (4× Laemmli dye (Bio-Rad) + 10% BME (VWR) and 50 μM biotin in wash buffer 1) was added to the beads. Proteins were eluted by heating at 95 °C for 5 min and run on a 4 to 20% precast gels (Bio-Rad). The lane was then excised and sent to the Biomedical Mass Spectrometry Center (University of Pittsburgh) for further purification and mass spectrometric analysis.

#### Mass spectrometry

Preparation of samples for mass spectrometry and the specifications used were as previously described (56).

#### V5 and His pull-downs

Transfected cells were treated with 20 μM MG-132 (Fisher Scientific) and 100 μM leupeptin (Sigma-Aldrich) for 2 h. Cells were isolated in lysis buffer (50 mM Tris, 1 mM ETDA, 0.5% Triton X-100, protease inhibitor (Thermo Scientific), 20 μM 1,10-phenanthroline (Sigma-Aldrich), 20 μM PR-619 (Sigma-Aldrich), 10 mM NEM (Sigma-Aldrich), and 150 mM NaCl in water). Twenty micromolar MG-132 and hundred micromolar leupeptin was added to 1.5-2 mg protein. Primary V5 antibody (Invitrogen, R960-25, 1:500 dilution) was added and samples rocked at RT for 1 h. Subsequently, we added protein A/G magnetic beads (Thermo Scientific) and rocked further at RT for 1 h. Samples were rinsed with wash buffer (50 mM Tris, 150 mM NaCl, 0.1% Triton X-100 in water) twice. 1× Laemmli buffer (Bio-Rad) was added to the beads and heated at 95 °C for 5 min. Beads were magnetically sorted prior to immunoblotting. For his pull-downs, cells were isolated in lysis buffer and His-tag proteins were isolated utilizing Dynabeads His-tag Isolation and Pull-down (Invitrogen) per manufacturer’s protocol.

#### Site-directed mutagenesis

Mutagenesis of lysine to arginine residues carried out by first designing forward and reverse primers (Integrated DNA Technologies) containing the desired point mutation. PCR was performed using a Phusion High-Fidelity DNA Polymerase (New England BioLabs) per manufacturer’s protocol. PCR products were digested with DpnI enzyme (NEB) at 37 °C for 2 h prior to transformation. Constructs were validated by sequencing.

#### Immunoprecipitations

Transfected cells were isolated in lysis buffer (1% Triton X-100, 0.2% SDS, 20 μM 1,10-phenanthroline (Sigma-Aldrich), 20 μM PR-619 (Sigma-Aldrich), 10 mM NEM (Sigma-Aldrich), and protease inhibitor (Thermo Scientific) in 1× PBS). Cells were collected and samples rotated in 4 °C for 10 to 15 min prior to sonication for 20 s at 25%. Between, 300 to 500 μg of total protein from cell lysates was precleared with protein A/G magnetic beads (Thermo Scientific) at 4 °C for 1 h. Primary V5 antibody (Invitrogen, R960-25, 1:500 dilution) was added and rotated for 1 h at RT. Protein A/G magnetic beads were added and then rotated for 2 h at RT. Beads were rinsed three times with wash buffer (0.5% Triton, 0.05% SDS, 1,10-phenanthroline, PR-619, NEM, and protease inhibitor in 1× PBS). 1× Laemmli buffer (Bio-Rad) was added to the beads and heated at 95 °C for 5 min. Beads were magnetically sorted prior to immunoblotting to remove the beads. Approximately, 20% of the eluant was loaded on the gel for the immunoblots.

#### Statistical analysis

Each experiment was repeated three times, unless otherwise indicated. Statistical comparisons were performed with means ± SD for continuous variables. All data were statistically analyzed by unpaired, 2-sample student’s *t* test to compare two independent groups, one-way ANOVA test was performed with post hoc Dunnett’s test for multiple comparisons, and two-way ANOVA test was performed to compare decay curves at multiple time points. *p* < 0.05 was considered statistically significant. All statistical analyses were performed with GraphPad Prism 9.

## Data availability

All data contained in this article will to be shared upon request.

## Supporting information

This article contains supporting information.

## Conflict of interest

The authors declare no conflicts of interest with the contents of this article.
