# Supplementary figures and images for: The E3 ligase subunit FBXO45 binds the interferon-λ receptor and promotes its degradation during influenza virus infection
Source: J Biol Chem. 2022 Nov 13;298(12):102698. doi: 10.1016/j.jbc.2022.102698 (PMC9747586; doi:10.1016/j.jbc.2022.102698)

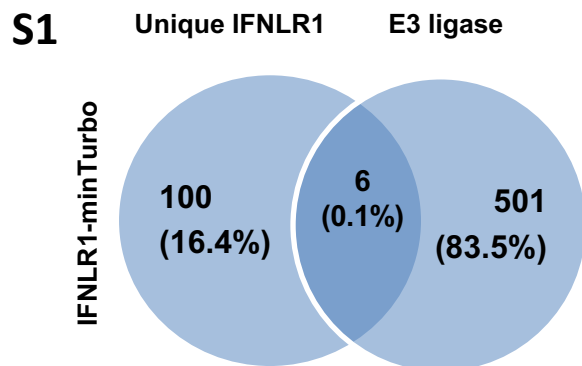

| IFNLR1 associated E3 Ligase |               |
|-----------------------------|---------------|
|                             | AFF4          |
|                             | <b>FBXO45</b> |
|                             | RNF10         |
|                             | SMURF2        |
|                             | USP53         |
|                             | ZFP91         |

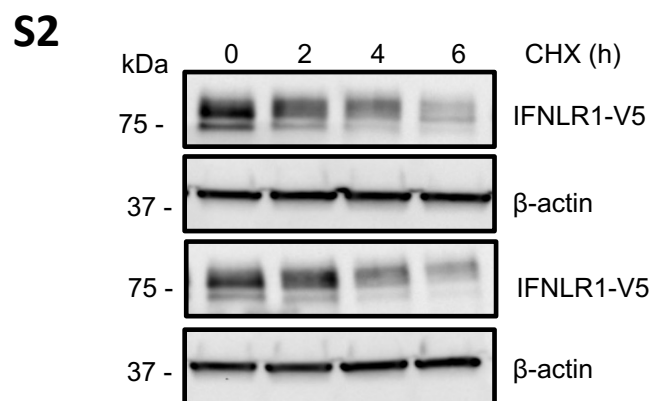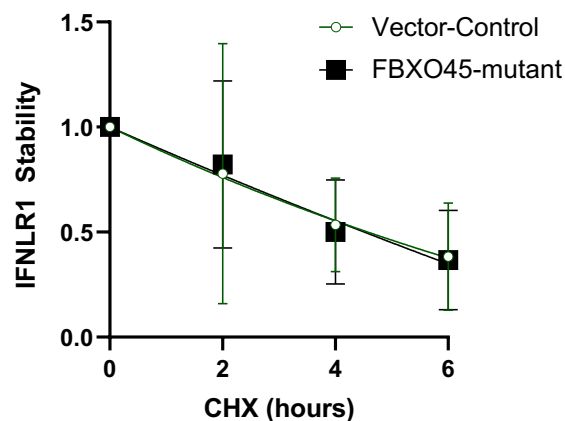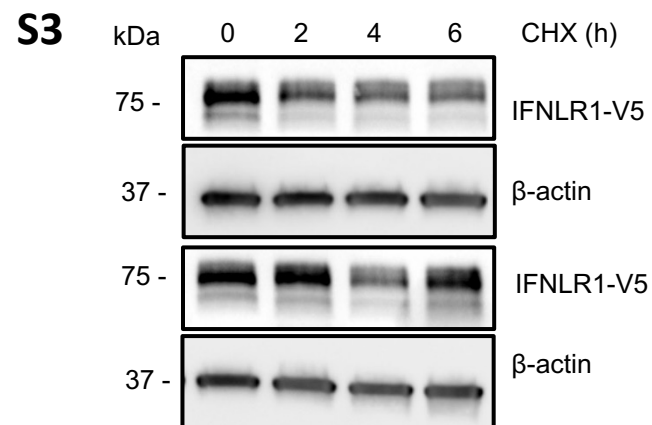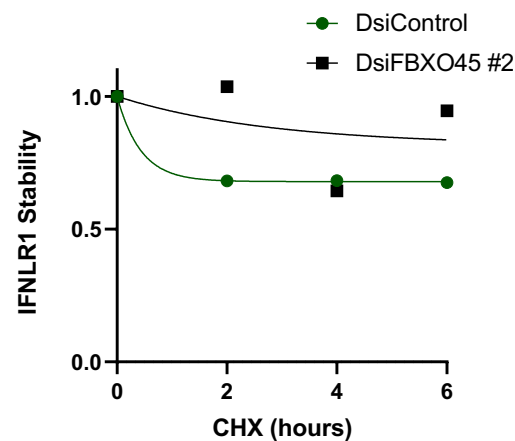

Supplement: Supplemental figures [file mmc1.pdf]
